# *Phoneutria nigriventer* Spider Toxin PnTx2-1 (δ-Ctenitoxin-Pn1a) Is a Modulator of Sodium Channel Gating

**DOI:** 10.3390/toxins10090337

**Published:** 2018-08-21

**Authors:** Steve Peigneur, Ana Luiza B. Paiva, Marta N. Cordeiro, Márcia H. Borges, Marcelo R. V. Diniz, Maria Elena de Lima, Jan Tytgat

**Affiliations:** 1 Toxicology and Pharmacology, University of Leuven (KU Leuven), Campus Gasthuisberg, P.O. Box 922, Herestraat 49, 3000 Leuven, Belgium; 2 Laboratório de Venenos e Toxinas Animais, Dept de Bioquímica e Imunologia, Instituto de Ciências Biológicas, Universidade Federal de Minas Gerais (UFMG), Belo Horizonte 31270-901, Brazil; mariaelena@santacasabh.org.br; 3 Departamento de Pesquisa e Desenvolvimento, Fundação Ezequiel Dias, Minas Gerais, Belo Horizonte 30510-010, Brazil; analubpaiva@gmail.com (A.L.B.P.); martadonascimento.phoneutria@gmail.com (M.N.C.); mhborgesb@gmail.com (M.H.B.); mdiniz@funed.mg.gov.br (M.R.V.D.); 4 Programa de Pós-graduação em Ciências da Saúde, Biomedicina e Medicina, Instituto de Ensino e Pesquisa da Santa Casa de Belo Horizonte, Grupo Santa Casa de Belo Horizonte, Minas Gerais, Belo Horizonte 31270-901, Brazil

**Keywords:** *Phoneutria nigriventer*, voltage-gated sodium channel, spider, insecticide, peptide toxin PnTx2-1, gating modifier toxin

## Abstract

Spider venoms are complex mixtures of biologically active components with potentially interesting applications for drug discovery or for agricultural purposes. The spider *Phoneutria nigriventer* is responsible for a number of envenomations with sometimes severe clinical manifestations in humans. A more efficient treatment requires a comprehensive knowledge of the venom composition and of the action mechanism of the constituting components. PnTx2-1 (also called δ-ctenitoxin-Pn1a) is a 53-amino-acid-residue peptide isolated from the venom fraction PhTx2. Although PnTx2-1 is classified as a neurotoxin, its molecular target has remained unknown. This study describes the electrophysiological characterization of PnTx2-1 as a modulator of voltage-gated sodium channels. PnTx2-1 is investigated for its activity on seven mammalian Na_V_-channel isoforms, one insect Na_V_ channel and one arachnid Na_V_ channel. Furthermore, comparison of the activity of both PnTx2-1 and PnTx2-6 on Na_V_1.5 channels reveals that this family of *Phoneutria* toxins modulates the cardiac Na_V_ channel in a bifunctional manner, resulting in an alteration of the inactivation process and a reduction of the sodium peak current.

## 1. Introduction

**Figure 1:**
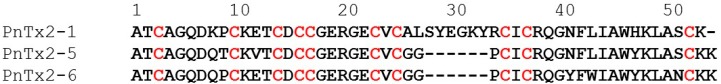
Sequence alignment of PnTx2-1 with Na_V_-channel toxins from *Phoneutria nigriventer* venom fraction 2.

Spiders can be considered one of the most successful venomous animals ever to inhabit the planet with over 47,000 species characterized to date [1]. Despite this diversity, no more than 100 spider species have had their venom investigated [2]. With over ten million estimated biologically active peptides in spider venoms [3], this means that less than 0.01% of spider peptides have been studied [3,4], making spider venom an untapped treasure of biologically active compounds with promising discoveries in drug and bioinsecticide development [1,5,6]. *Phoneutria nigriventer* are very aggressive, solitary spiders. They are active hunters, relying on their fast-acting and efficient venom for prey capture and defense. They prey normally on insects, although there are reports of *Phoneutria* hunting on other spiders and small rodents as well [6,7]. Human envenomation by *Phoneutria nigriventer* is common in Brazil, with 0.5–1% resulting in severe envenomation, with most of these occurring in children [8]. Cysteine-rich peptide toxins with action on ion channels are the most abundant components in its venom [9]. A recent review describes the complexity of this venom [6]. Historically, the *Phoneutria* toxins are annotated based on their occurrence in the venom when following the venom purification methods used in the first studies [10], that is, based on a particular chromatographic step and in the order of elution of the toxin, in this step. However, a new nomenclature has been proposed based on the genus of the spider, the target of the toxin and the isoform [11]. According to this nomenclature, PnTx2-1 is also called δ-ctenitoxin-Pn1a and it can be accessed in the Arachnoserver databank (http://www.arachnoserver.org) [2]. In-vivo studies have shown that the PhTx2 fraction is toxic to both mice and insects [12]. Nine peptides could be identified from the PhTx2 fraction. These peptides were named accordingly PnTx2-1 to PnTx2-9. PnTx2-1, PnTx2-5 and PnTx2-6 showed higher toxicity after intracerebral (i.c.) injection in mice [13]. Both PnTx2-5 and PnTx2-6 have been characterized as modulators of the inactivation of voltage-gated sodium (Na_V_) channels [14,15]. Toxin PnTx2-1 (5838.8 Da) shares up to 77% identity with PnTx2-5 and PnTx2-6 (Figure 1) [13]. When injected in mice, PnTx2-1 causes pruritus, lacrimation, increased salivation, sweating and agitation followed by spastic paralysis of the limbs [12]. A recent transcriptomic and proteomic investigation of *P. nigriventer* venom showed that PnTx2-1, altogether with its isoforms, is among the most expressed toxins in this venom [9], suggesting that this toxin may play an important role in the envenoming of natural preys. Based on the in-vivo tests in mice and the sequence homology with PnTx2-5 and PnTx2-6, this peptide is classified as a Na_V_-channel toxin. However, it has never been tested on any Na_V_-channel isoform. Therefore, in this study the Na_V_-channel subtype selectivity and species specificity of PnTx2-1 are investigated. Furthermore, comparison of the activity of both PnTx2-1 and PnTx2-6 on Na_V_1.5 reveals that this family of *Phoneutria* toxins modulates the cardiac Na_V_ channel in a bifunctional manner, resulting in an alteration of the inactivation process and a reduction of the sodium peak current.

## 2. Results

### 2.1. Electrophysiological Charaterization

#### 2.1.1. Activity of PnTx2-1 on Mammalian Na_V_ Channels

**Figure 2:**
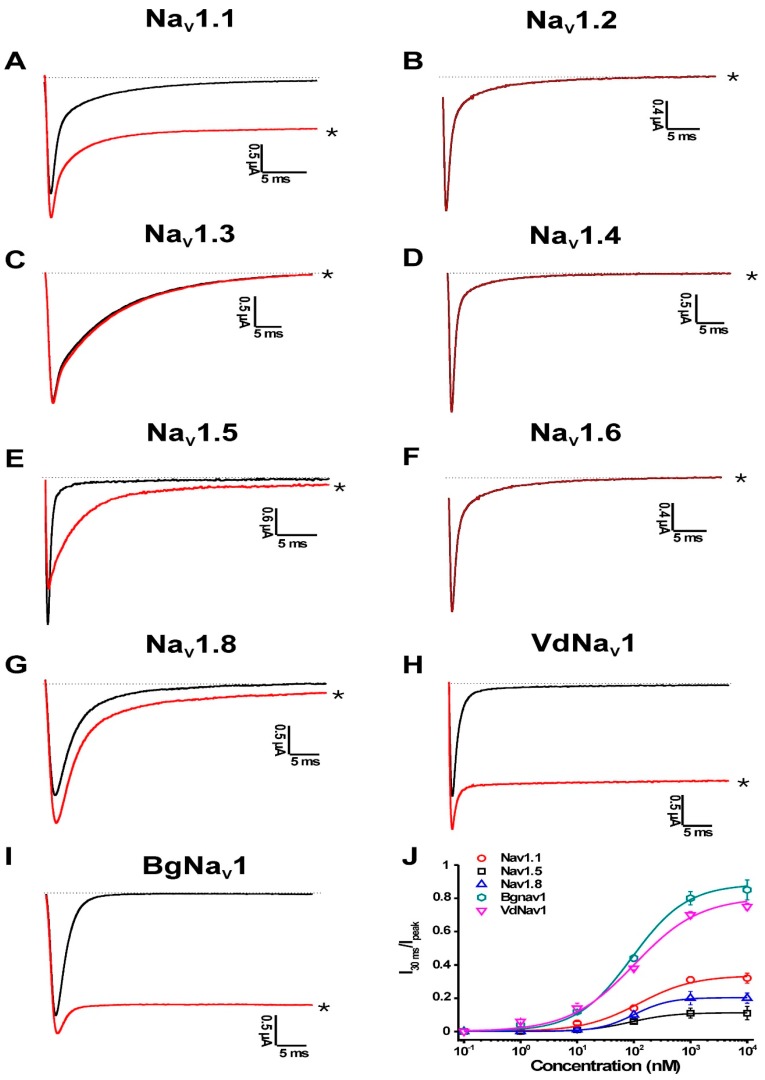
Electrophysiological profiles of PnTx2-1 on Na_V_s. Panels show superimposed current traces of 1 μM PnTx2-1. The dotted line indicates zero current level. Black, current trace in control conditions; red, current trace in toxin situation. The asterisk marks steady-state current trace after application of 1 µM peptide. The last panel shows the concentration–response curves for PnTx2-1 on different Na_V_ channel isoforms.

PnTx2-1 was screened against a panel of seven mammalian Na_V_ channel isoforms (Na_V_1.1–Na_V_1.6 and Na_V_1.8), one insect from the cockroach *Blattella germanica* (BgNa_V_1) and one arachnid channel from the mite *Varroa destructor* (VdNa_V_1) (Figure 2). PnTx2-1 was investigated for its activity on these Na_V_ channels because of their ability to be effectively expressed in oocytes, and these are the most commonly screened Na_V_ channels when testing for Na_V_ channel effects of peptide toxins [16].

**Figure 3:**
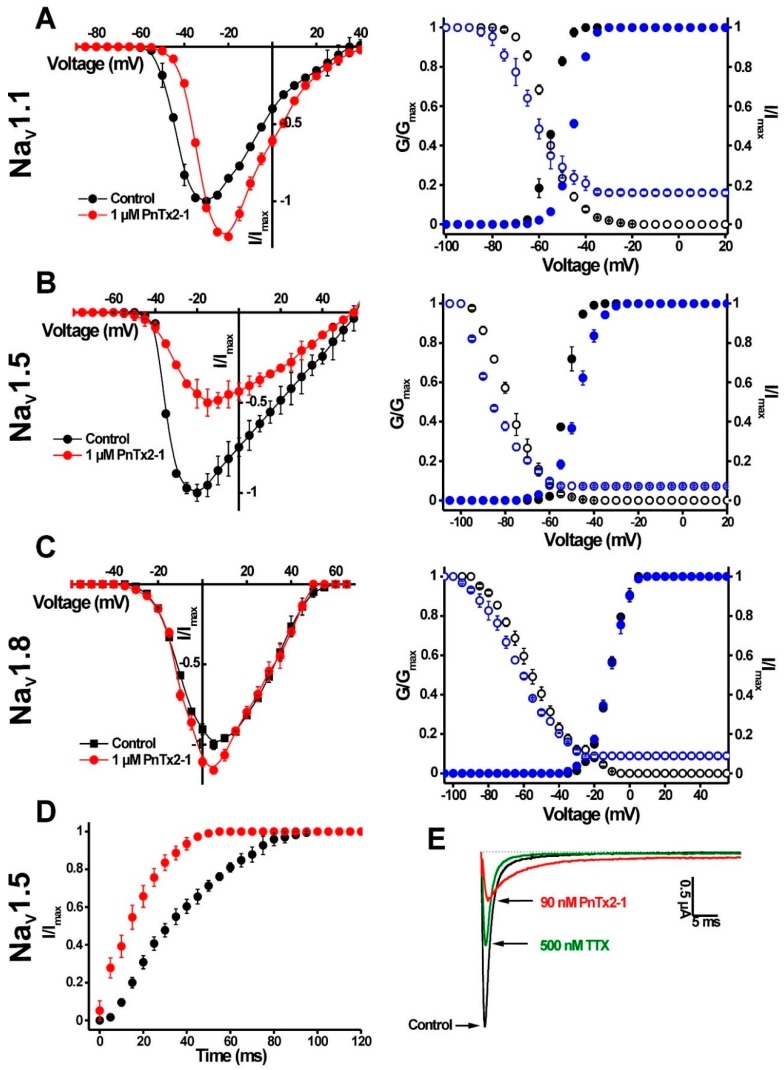
Electrophysiological characterization of PnTx2-1 on mammalian Na_V_ channels. Left panels show the current voltage relationships and the right panels show the steady-state activation (closed symbols) and inactivation (open symbols) curves in control (black) and toxin conditions (1 µM PnTx2-1, blue) for Na_V_1.1 (**A**), Na_V_1.5 (**B**) and Na_V_1.8 (**C**). (**D**) Recovery from inactivation for Na_V_1.5 channels. Control conditions (black symbols) and in the presence of 1 µM PnTx2-1 (red symbols) are shown. (**E**) Competitive experiments to indicate that PnTx2-1 does not bind at site 1. Representative traces for Na_V_ 1.5 channels are shown in control; after application of 500 nM tetrodotoxin (TTX) and after subsequent addition of 90 nM PnTx2-1.

Among the mammalian isoforms, 1 μM PnTx2-1 slowed the inactivation of Na_V_1.1, Na_V_1.5 and Na_V_1.8. Na_V_1.2, Na_V_1.3, Na_V_1.4 and Na_V_1.6 were not affected by PnTx2-1. Interestingly, PnTx2-1 had a profound effect on the inactivation of the insect channel BgNa_V_1 and the arachnid channel VdNa_V_1. PnTx2-1 completely inhibited inactivation of BgNa_V_1 channels, resulting in sustained non-inactivating currents. Concentration–response curves were constructed to determine the values at which half of the channels were modulated by PnTx2-1. Among the mammalian isoforms, the half-maximal effective concentration (EC_50_) values yielded 122.1 ± 34.6 nM, 87.0 ± 7.5 nM and 101.1 ± 5.1 nM for Na_V_1.1, Na_V_1.5 and Na_V_1.8, respectively. Activation and steady-state inactivation curves were constructed for Na_V_1.1, Na_V_1.5 and Na_V_1.8 channels (Figure 3A–C, Table 1). In the presence of 1 µM toxin, a significant modulation of gating kinetics was observed for Na_V_1.1 and Na_V_1.5 (Figure 3A,B, Table 1). For both Na_V_1.1 and Na_V_1.5 channels, the midpoint of activation shifted towards more depolarized potentials. At the same time, the steady-state inactivation curves shifted towards more hyperpolarized membrane potentials. This resulted in an increased window of open probability for both Na_V_1.1 and Na_V_1.5. No relevant modulation of the gating kinetics was observed for Na_V_1.8-channel isoforms since no significant shift in the half-maximal activation voltage (V_1/2_) values was noted (Figure 3C, Table 1). No significant change in the reversal potential was observed in the presence of toxin which indicates that the ion selectivity of Na_V_ channels is not altered. One micromolar PnTx2-1 strongly enhanced the recovery from inactivation for Na_V_1.5 channels with τ = 50.2 ± 3.0 ms in control conditions and τ = 22.8 ± 3.2 ms in the presence of the toxin (Figure 3D). To verify whether PnTx2-1 binds to neurotoxin site 1, competitive binding experiments were performed. Application of tetrodotoxin (TTX) at its half-maximal inhibitory concentration (IC_50_) resulted in a blockage of half of the expressed Na_V_1.5 channels. This was observed as a 50% decrease of the sodium current peak amplitude. An additional reduction of the peak amplitude was the result of subsequent and additional application of PnTx2-1 at its EC_50_ (*n* = 6) (Figure 3E).

#### 2.1.2. Activity of PnTx2-1 on Insect Na_V_-Channel Currents

**Figure 4:**
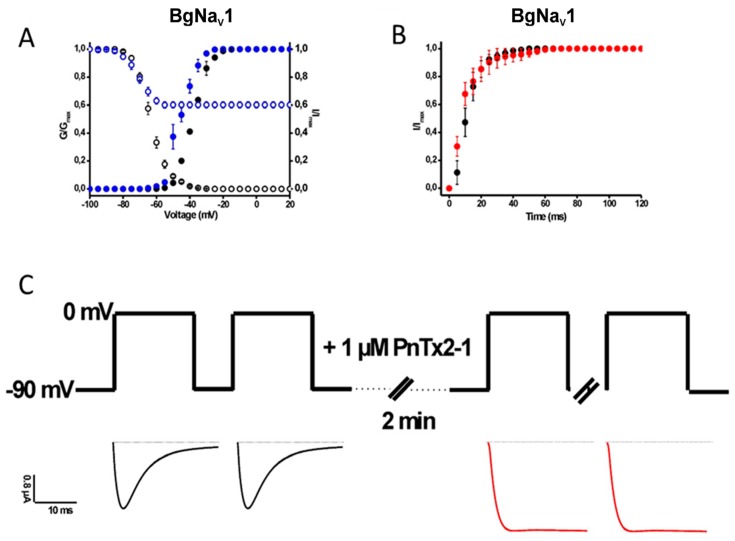
Electrophysiological characterization of PnTx2-1 on insect Na_V_ channels. (**A**) Steady-state activation (closed symbols) and inactivation (open symbols) curves in control (black) and toxin conditions (1 µM PnTx2-1, blue) for BgNa_V_1.1. (**B**) Recovery from inactivation in control (black symbols) and in the presence of 1 µM PnTx2-1 (red symbols). (**C**) Investigation of the state-dependence of indicating that an expected degree of channel inactivation inhibition was observed after the 2 min incubation, indicating that the open state is not required for toxin interaction with the channel.

The EC_50_ values for BgNa_V_1 and VdNa_V_1 were determined at 91.9 ± 17.0 nM and 101.8 ± 14.7 nM, respectively. The insect Na_V_ channel BgNa_V_1 was used to further investigate the characteristics of PnTx2-1 in inducing modulation of channel gating (Figure 4). A shift in the midpoint of activation and in the voltage dependence of steady-state inactivation was observed (Table 1). From Figure 4A, it can be seen that the steady-state inactivation became incomplete after toxin addition. A 62.3 ± 3.2% non-inactivating current component of steady-state inactivation can be seen. In addition, we examined the recovery from fast inactivation in the absence or presence of PnTx2-1 and found that 1 µM does not influence the recovery from fast inactivation in BgNa_V_1 (Figure 4B). Considering the incomplete steady-state inactivation of BgNa_V_1 channels in the presence of PnTx2-1, we decided to determine whether inhibition occurs during the closed or open state of the channel. One micromolar toxin was applied to the bath solution with oocytes clamped at −90 mV, allowing interaction with the membrane for 2 min without depolarizing the membrane. After 2 min, currents were elicited by a depolarizing pulse to 0 mV. The obtained currents in the presence of toxin were normalized to the currents obtained in the same cells in control conditions. A significant slowing down of inactivation was observed when PnTx2-1 was applied to the oocytes without applying depolarizing pulses (Figure 4C). This indicates that there is toxin interaction with the channel in the closed state and suggests that membrane depolarization and hence most likely channel opening is not required to allow the toxin to bind.

#### 2.1.3. Activity of PnTx2-6 on Na_V_1.5-Channel Currents

**Figure 5:**
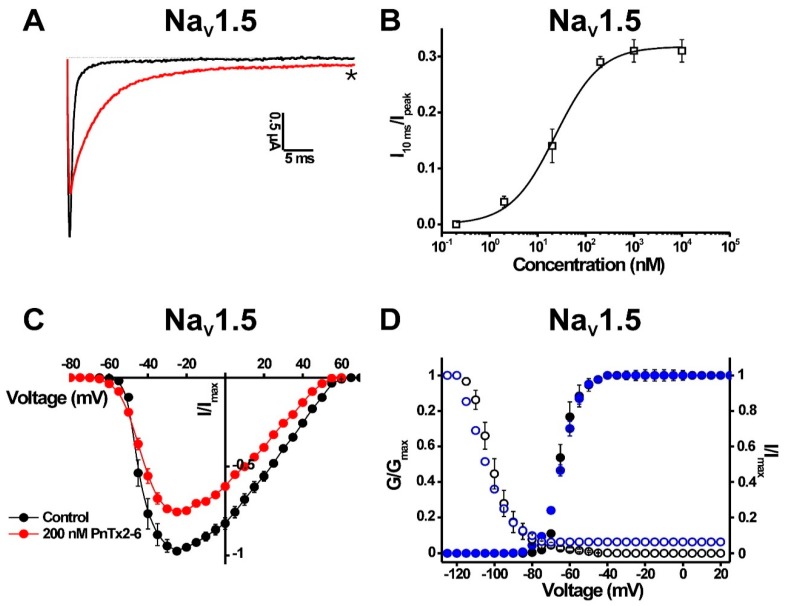
Electrophysiological characterization of PnTx2-6 on Na_V_1.5. (**A**) Representative whole-cell current traces in control (black) and toxin (red) conditions are shown. The dotted line indicates the zero-current level. The asterisk marks steady-state current trace after application of 1 µM peptide. (**B**) Concentration–response curve for PnTx2-6 on Na_V_1.5. (**C**) Current–voltage relationship in control conditions and in the presence of 200 nM PnTx2-6. (**D**) Steady-state activation (closed symbols) and inactivation (open symbols) curves in control (black) and toxin conditions (1 µM PnTx2-6, blue) for Na_V_1.5 channels.

The pharmacological phenotype induced on Na_V_1.5 channels by PnTx2-1 is very similar to what has been reported previously for PnTx2-6 [17]. Therefore, we performed further characterisation of PnTx2-6 on Na_V_1.5 channels by investigating the current–voltage relationship in the presence of PnTx2-6. In the presence of 1 µM PnTx2-6, an inhibition of the sodium peak current and a delay of the inactivation was observed (Figure 5A). The slowing down of inactivation was characterized by an EC_50_ value of 22.3 ± 3.1 nM (Figure 5B). The voltage–current relationship shows a reduction of the sodium current by 21.3 ± 2.8% in the presence of 200 nM PnTx2-6 (Figure 5C). Construction of the activation and steady-state inactivation curves indicated that no significant modification of gating processes occurs (Figure 5D).

## 3. Discussion

PnTx2-1 shares the highest sequence identity with PnTx2-5 and PnTx2-6, which are also isolated from the *Phoneutria nigriventer* venom fraction 2 [13]. PnTx2-5 and PnTx2-6 are the best-characterized toxins from the PhTx2 venom fraction [14,15,17,18]. These two toxins share 90% sequence identity, with only five amino acid residues different (Figure 1). PnTx2-5 and PnTx2-6 are responsible for the painful and persistent penile erection, also known as priapism, a common clinical manifestation observed upon *Phoneutria nigriventer* envenomation [18,19]. Both peptides modulate sodium channel kinetics by slowing down the inactivation process and by shifting the voltage dependence of activation towards more hyperpolarized potentials [14,15]. Interestingly, significant differences in potency have been reported despite the high homology between both peptides. PnTx2-5 displayed an approximately six-fold lower potency than PnTx2-6 on macroscopic sodium currents in patch clamp experiments using GH3 cells [20]. It has been suggested that Tyr41 and Trp43 in PnTx2-6 are involved in the higher potency compared to PnTx2-5 [14]. Interestingly, these residues are also not conserved in PnTx2-1 (Figure 1). Nevertheless, similar EC_50_ values were found for PnTx2-1 and PnTx2-6 on Na_V_1.5 channels (Figure 2J and Figure 5B). While PnTx2-6 exerts a strong affinity for Na_V_1.2–Na_V_1.4 and Na_V_1.6 channels [17], PnTx2-1 shows no activity on these channels, even at concentrations up to 5 µM (data not shown). It is well established that even minor differences in amino acid composition of peptide toxins can influence the Na_V_ channel isoform selectivity [21,22]. Binding experiments in brain synaptosomes indicated a partial competition between the α-scorpion toxin AaHII (from *Androctonus australis Hector*) and PnTx2-6 [14]. It thus can be hypothesized that PnTx2-1 also acts by binding to a site similar to the site that PnTx2-6 and the typical α-scorpion toxins bind to. β-Scorpion toxins and certain spider toxins modulate Na_V_ channels by shifting the midpoint of activation towards more negative potentials, often resulting in a reduction in sodium peak current [23,24,25]. However, the β-scorpion toxin CssIV (from *Centruroides suffusus suffusus*) does not compete with PnTx2-6. Therefore, it seems unlikely that PnTx2-6 shares a binding site with β-scorpion toxins [14]. However, it can be suggested that the PnTx2-1- and PnTx2-6-induced inhibition of the sodium peak current (Figure 2E, Figure 3B and Figure 5A) results from gating modification, rather than physical obstruction of the ion pathway. Scorpion toxins such as Ts1 (from *Tityus serrulatus*) and Tz1 (from *Tityus zulianus*) induce a similar pharmacological phenotype activity on Na_V_1.5 channels to that observed for PnTx2-1 [23,25,26]. For these toxins, it was evidenced that by trapping the voltage sensor in the inward position, channels are prevented from opening, which is seen as an inhibition of the sodium current for Na_V_1.5 channels [16,23,25,26].

δ-Atracotoxins (δ-ACTX) are a family of Na_V_ channel-targeting toxins isolated from the venom of Australian funnel-web spiders [24]. They are classified in the Na_V_ channel-targeting spider toxin (NaSpTx) family 4. This family constitutes six peptides isolated from Australian funnel-web spiders and one peptide from the Asian spider *Macrothele gigas*. They are peptides of 42 amino acid residues including eight conserved cysteine residues which form four disulfide bridges in an inhibitor cysteine-knot (ICK) motif [24,27]. Although very low sequence homology (<25%) can be found between PnTx2-1 and these funnel-web spider toxins, they do seem to modulate the Na_V_ channels in a similar way. In patch-clamp experiments, it was shown that δ-ACTX interact with TTX-sensitive but not with TTX-resistant sodium currents in dorsal root ganglion (DRG) neurons. To date, no information is available on the Na_V_-channel subtype selectivity of δ-ACTX since these peptides have not yet been tested on heterologously expressed Na_V_-channel isoforms. However, in DRGs, δ-ACTX produce a selective slowing of Na_V_-current inactivation and a reduction in peak sodium current. Moreover, they cause a hyperpolarizing shift in the midpoint of activation, and an increased recovery from inactivation was seen for toxin-bound channels [20,28,29,30]. Radiolabeled binding assays have determined that the δ-ACTX bind to the neurotoxin site 3 [31]. Several residues have been suggested to be important for the interaction of δ-ACTX with the Na_V_ channels. However, this family still awaits structure–activity data to elucidate the exact key residues [4,24]. It thus seems that PnTx2-1 and the δ-ACTX exert a comparable complex voltage-dependent modulation of Na_V_ channel gating.

Although both toxins were insecticidal to the larval and adult forms of the housefly [13], PnTx2-5 and PnTx2-6 have never been evaluated for their insecticidal activity towards insect Na_V_ channels. In fact, it was always believed that *Phoneutria nigriventer* venom fraction 4 (PhTx4) was the venom fraction with the highest and most specific insecticidal activity [6,32]. However, here we show that peptides of PhTx2 fraction have a similar insecticidal potential, at least regarding Na_V_ channels. PnTx2-1 is the first member of this spider toxin family to be characterized in depth for its activity on insect Na_V_ channels. The weak species selectivity of PnTx2-1 challenges the suitability of this peptide for the development of novel insecticidal agents.

## 4. Conclusions

The characterization of PnTx2-1 as a Na_V_-channel modulator adds to the expanding knowledge on the *Phoneutria nigriventer* venom. A better understanding of the mechanism of action of its composing venom peptides is important for an effective treatment of *Phoneutria nigriventer* envenomation. This work reports for the first time the activity of a toxin isolated from the *Phoneutria nigriventer* venom fraction 2 (PhTx2) on insect Na_V_ channels. This observation indicates that besides venom fraction 4, fraction 2 might also be considered as a source of insecticidal peptides.

## 5. Materials and Methods

### 5.1. Toxin Purification

Crude venom from *P. nigriventer* was collected from mature male and female spiders maintained in the Scientific Arachnidium at the Fundacion Ezequiel Dias (FUNED) in Belo Horizonte, Brazil (Sisgen: License for Access to Genetic Patrimony Number 010815/2015-5). Toxin PnTx2-1 was purified as previously described [13].

### 5.2. Electrophysiology

#### 5.2.1. Heterologous Expression in *Xenopus laevis* oocytes

For expression in *X. laevis* oocytes, the plasmids encoding the α-subunits Na_V_1.1–Na_V_1.6, Na_V_1.8, cockroach *Blattella germanica* BgNa_V_1.1, *Varroa destructor* VdNa_V_1, and the corresponding β-subunits, rβ1, hβ1 and *Drosophila melanogaster* TipE, were linearized with the respective restriction enzymes, mentioned between parentheses, and transcribed using the T7 or SP6 mMESSAGE-mMACHINE transcription kit (Ambion, Austin, TX, USA). Stage V and VI oocytes were harvested from anesthetized female *X. laevis* frogs, as described previously [33]. Oocytes were injected with 30–50 nL of 1–3 μg/μL Na_V_-channel cRNA using a microinjector (Drummond Scientific, Broomall, PA, USA). The oocytes were then incubated in ND96 solution (in mM: NaCl 96, KCl 2, MgCl_2_ 1, CaCl_2_ 1.8, HEPES 5), adjusted to pH 7.5 and supplemented with 50 mg/L of gentamycin sulphate and 90 mg/L theophylline, at 16 °C for 1–5 days, until expression of ion channels.

#### 5.2.2. Electrophysiological Recordings

Electrophysiological recordings were performed as described previously [33]. In brief, whole-cell currents from oocytes were recorded at room temperature (18–22  °C) by the two-electrode voltage clamp technique using a GeneClamp 500 amplifier (Molecular Devices, Sunnyvale, CA, USA) controlled by a pClamp data acquisition system (Molecular Devices). Oocytes were placed in a bath containing ND96 solution. Voltage and current electrodes were filled with 3  M KCl, and the resistances of both electrodes were kept between 0.7 and 1.5 MΩ. The elicited currents were sampled at 20  kHz and filtered at 2  kHz using a four-pole, low-pass Bessel filter. To eliminate the effect of the voltage drop across the bath grounding electrode, the bath potential was actively controlled by a two-electrode bath clamp. Leak subtraction was performed using a −P/4 protocol. Whole-cell current traces were evoked every 5  s by a 100-ms depolarization to the voltage corresponding to the maximal activation of the Na_V_ subtype in control conditions, starting from a holding potential of −90 mV. The oocyte expressing the specific Na_V_-channel isoform with no toxin added to the bath was considered as control. Concentration–response curves were constructed by adding different toxin concentrations directly to the bath solution. The percentage of Na_V_ modulation was plotted against the logarithm of the applied concentrations and fitted with the Hill equation:

y  =  100/[1  +  (IC_50_/[toxin])] × h (1)

where y is the amplitude of the toxin-induced effect, IC_50_ is the toxin concentration at half-maximal efficacy, [toxin] is the toxin concentration and h is the Hill coefficient. The amplitude of the toxin-induced effect is obtained by dividing the current amplitude at 30 ms in steady-state toxin situation by the peak current amplitude in control conditions. To investigate the effects on the voltage dependence of activation, current traces were induced by 100-ms depolarizations from a holding potential of −90 to 65  mV with 5-mV increments. To investigate the effects on the steady-state inactivation process, oocytes were depolarized using a standard two-step protocol. From a holding potential of −90 mV, 100-ms prepulses were generated, ranging from −90 to 65  mV with 5-mV increments, immediately followed by a 100-ms test pulse to 0  mV. Data were normalized to the maximal Na^+^ current amplitude, plotted against prepulse potential and fitted using the Boltzmann equation:

I_Na_/I_max_  =  [(1  −  C)/(1  +  exp ((V  −  Vh)/k))]  +  C (2)

where I_max_ is the maximal I_Na_, Vh is the voltage corresponding to half-maximal inactivation, V is the test voltage, k is the slope factor, and C is a constant representing a non-inactivating persistent fraction (close to zero in control). All data were tested for normality using a D’Agustino Pearson omnibus normality test. Data following a Gaussian distribution were analyzed for significance using one-way ANOVA and the Bonferroni test. Nonparametric data were analyzed for significance using the Kruskal–Wallis and Dunn tests. All data were analyzed using pClamp Clampfit 10.4 (Molecular Devices^®^, Downingtown, PA, USA, 2003) and Origin 7.5 software (Originlab^®^, Northampton, MA, USA, 2003) and are presented as mean  ±  standard error (SEM) of at least 3 independent experiments (*n*  ≥  3).

## Figures and Tables

**Table 1:** V_1/2_ values for the activation and steady-state inactivation curves obtained for Na_V_1.1, Na_V_1.5, Na_V_1.8 and BgNa_V_1.

	Activation		Inactivation	
V_1/2_ (mV)	Control	PnTx2-1 (1 µM)	Control	PnTx2-1 (1 µM)
Na_V_1.1	−56.4 ± 0.2	−63.4 ± 0.1	−54.7 ± 0.1	−45.3 ± 0.1
Na_V_1.5	−53.1 ± 0.1	−45.3 ± 0.1	−78.4 ± 0.2	−87.5 ± 0.7
Na_V_1.8	−11.5 ± 0.1	−11.4 ± 0.1	−55.8 ± 0.4	−63.9 ± 0.4
BgNa_V_1	−38.1 ± 0.1	−45.8 ± 0.3	−63.3 ± 0.3	−70.7 ± 0.13
